# Synthesis, Characterization, Cytotoxic Activity, and Interactions with CT-DNA and BSA of Cationic Ruthenium(II) Complexes Containing Dppm and Quinoline Carboxylates

**DOI:** 10.1155/2017/2562780

**Published:** 2017-07-26

**Authors:** Edinaldo N. da Silva, Paulo A. B. da Silva, Angélica E. Graminha, Pollyanna F. de Oliveira, Jaqueline L. Damasceno, Denise C. Tavares, Alzir A. Batista, Gustavo Von Poelhsitz

**Affiliations:** ^1^Instituto de Química, Universidade Federal de Uberlândia, 38400-902 Uberlândia, MG, Brazil; ^2^Departamento de Química, Universidade Federal de São Carlos, 13565-905 São Carlos, SP, Brazil; ^3^Universidade de Franca, 14404-600 Franca, SP, Brazil

## Abstract

The complexes* cis*-[Ru(quin)(dppm)_2_]PF_6_ and* cis*-[Ru(kynu)(dppm)_2_]PF_6_ (quin = quinaldate; kynu = kynurenate; dppm =* bis*(diphenylphosphino)methane) were prepared and characterized by elemental analysis, electronic, FTIR, ^1^H, and ^31^P{^1^H} NMR spectroscopies. Characterization data were consistent with a* cis* arrangement for the dppm ligands and a bidentate coordination through carboxylate oxygens of the quin and kynu anions. These complexes were not able to intercalate CT-DNA as shown by circular dichroism spectroscopy. On the other hand, bovine serum albumin (BSA) binding constants and thermodynamic parameters suggest spontaneous interactions with this protein by hydrogen bonds and van der Waals forces. Cytotoxicity assays were carried out on a panel of human cancer cell lines including HepG2, MCF-7, and MO59J and one normal cell line GM07492A. In general, the new ruthenium(II) complexes displayed a moderate to high cytotoxicity in all the assayed cell lines with IC_50_ ranging from 10.1 to 36 *µ*M and were more cytotoxic than the precursor* cis*-[RuCl_2_(dppm)_2_]. The* cis*-[Ru(quin)(dppm)_2_]PF_6_ were two to three times more active than the reference metallodrug cisplatin in the MCF-7 and MO59J cell lines.

## 1. Introduction

The disseminated use of cisplatin and other platinum based metallodrugs as chemotherapeutic agents against ovarian, bladder, and testicular cancers, among others, is still a key aspect for the development of the medicinal inorganic chemistry [1–4]. In the search for coordination compounds active against tumors and less toxic than cisplatin, ruthenium compounds emerge as the most promising with biological features including mechanism of action, toxicity, and biodistribution which are very different from those of classical platinum compounds and might therefore be active against resistant human cancers [3, 5–8]. In the last years three ruthenium(III) complexes entered clinical trials: NAMI-A - [ImH][*trans*-RuCl_4_(DMSO)(Im)], KP1019 - [InH][*trans*-RuCl_4_(In)_2_], and NKP3019 - Na[*trans*-RuCl_4_(In)_2_] (In = indazole) [7, 9–12]. For recent developments on the anticancer activity of these ruthenium(III) complexes see cited references.

Previous work from our group displayed biological results from the diphosphonic ruthenium(II) precursors* cis*-[RuCl_2_(P-P)_2_], P-P = dppm or dppe, and its derivatives with 2-pyridinecarboxylic acid anion (pic), the* cis*-[Ru(pic)(P-P)_2_]PF_6_, with an* -N,O* chelation for the pic ligand [13, 14]. The antimycobacterial activity against MTB H_37_Rv indicated a MIC value of 26.6 *µ*M for the precursor and a much higher activity for the* cis*-[Ru(pic)(dppm)_2_]PF_6_ with a MIC value of 0.69 *µ*M [13]. Some additional studies performed with the analogous* cis*-[Ru(pic)(dppe)_2_]PF_6_ revealed a high antibacterial activity against* S. aureus*,* C. albicans,* and* M. smegmatis* with MIC in the range 0.3 to 5.3 *µ*M [14]. In the assay of acute oral toxicity this complex belongs to class 5 (a substance with LD_50_ greater than 2000 and less than 5000 mg·kg^−1^ body weight), indicating a relatively low acute toxicity [14]. Species containing bidentate carboxylates such as* cis*-[Ru(dicl)(dppm)_2_]PF_6_,* cis*-[Ru(ibu)(dppm)_2_]PF_6_ and* cis*-[Ru(prop)(dppe)_2_]PF_6_ were studied and presented moderate to high cytotoxic activity against human cancer cell lines [15, 16].

Due to this background of promising biological results in complexes containing the* cis*-[Ru(P-P)_2_] unit our current strategy consists in evaluating other derivatives with different chelating moiety replacing the chlorido ligands in the search for new cytotoxic agents against tumor cells. In the current work quinoline carboxylates were chosen as coligands in order to generate cationic complexes and also to explore the possible different coordination modes of the ligands.

In this work the synthesis and characterization of two new derivatives with formula* cis*-[Ru(quin)(dppm)_2_]PF_6_** (1)** and* cis*-[Ru(kynu)(dppm)_2_]PF_6_** (2)** are reported. The interaction of these complexes with CT-DNA and BSA was investigated by circular dichroism (CD) and fluorescence spectroscopies, respectively. Besides, preliminary in vitro tests of cytotoxic activities against a variety of human cell lines are presented and discussed.

## 2. Experimental

### 2.1. Chemicals

Solvents were purified by standard methods. All chemicals used were of reagent grade or comparable purity. The RuCl_3_·3H_2_O and the ligands 1,1-*bis*(diphenylphosphino)methane (dppm), quinaldic acid, and kynurenic acid were used as received from Aldrich. The* cis*-[RuCl_2_(dppm)_2_] precursor complex was prepared according to the literature method [17].

### 2.2. Instrumentation

Elemental analyses were performed on a Perkin Elmer 2400 Series II CHNS/O microanalyser. Molar conductivities of freshly prepared 1.0 × 10^−3^ mol·dm^−3^ methanol solutions were measured using a Digimed DM-31 conductivity meter. IR spectra were recorded on a Shimadzu FTIR-Prestige 21 spectrophotometer, using KBr pellets. UV-vis spectroscopy was recorded on a Femto model 800 XI spectrophotometer using cuvettes of 1 cm path length. ^1^H and ^31^P{^1^H} NMR experiments were performed on a Bruker Avance III HD 400 MHz (9.4 T) at 298 K. Spectra were recorded in CDCl_3_ with TMS and 85% H_3_PO_4_ as external references, respectively, for ^1^H and ^31^P{^1^H}.

### 2.3. Synthesis

The precursor* cis*-[RuCl_2_(dppm)_2_] (0.103 mmol; 100 mg) was solubilized in 100 mL of methanol and the mixture was heated during 20 minutes. Quinaldic acid (0.103 mmol; 17.8 mg) or kynurenic acid (0.103 mmol; 19.5 mg), respectively, for synthesis of complexes** 1** and** 2**, was solubilized in 10 mL of methanol and deprotonated with triethylamine (0.132 mmol; 0.014 ml) and this solution was added dropwise on the precursor solution. After this processes, the mixture was stirred and refluxed for 48 h. The final solution was concentrated to ca. 5 mL and an aqueous solution of NH_4_PF_6_ (0.150 mmol; 24.4 mg) was added for the precipitation of a yellow solid. The solid was filtered off and washed with water (3 × 5 mL) and diethyl ether (3 × 5 mL) and dried under reduced pressure.

#### 2.3.1. *cis*-[Ru(quin)(dppm)_2_]PF_6_** (1)**

Yield: 79.1 mg (63%). Anal. Calcd for C_60_H_50_F_6_NO_2_P_5_Ru: exptl (calc) C, 60.97 (60.71); H, 4.93 (4.63); N, 1.10 (1.18). ^1^H NMR (CDCl_3_): *δ* 4.11, 4.73 (m × 2, 2 × 2H; PCH_2_P), 6.26 (m, 4H; C_6_H_5_), 6.99–7.86 (m, 39H; C_6_H_5_ + quin), 7.91 (d, *J*_HH_ = 8.1 Hz, 1H; quin), 8.22 (d, *J*_HH_ = 8.5 Hz, 1H; quin), 8.31 (d, *J*_HH_ = 8.5 Hz, 1H; quin) ppm. ^31^P{^1^H} NMR (161.73 MHz - CDCl_3_): *δ* 9.70 (t, 2P, *J*_PP_ = 39 Hz); –12.2 (t, 2P, *J*_PP_ = 39 Hz); –144.7 (sept, 1P, *J*_PF_ = 711 Hz). UV-vis (CH_2_Cl_2_, 5.0 × 10^–5^ M): *λ*/nm (*ε*/M^–1^ cm^–1^) 256sh (4.33 × 10^4^), 325 (8.00 × 10^3^). Molar conductivity [Λ_M_/(S·cm^2^·mol^–1^) in methanol: 79.0 (range for a 1 : 1 electrolyte: 80–115) [18].

#### 2.3.2. *cis*-[Ru(kynu)(dppm)_2_]PF_6_** (2)**

Yield: 73.0 mg (57%). Anal. Calcd for C_60_H_50_F_6_NO_3_P_5_Ru: exptl (calc) C, 59.87 (59.91); H, 4.68 (4.39); N, 1.14 (1.16). ^1^H NMR (CDCl_3_): *δ* 4.14, 4.73 (m × 2, 2 × 2H; PCH_2_P), 6.26 (m, 4H; C_6_H_5_), 6.80 (s, 1H; kynu), 6.96–7.77 (m, 39H; C_6_H_5_ + kynu), 8.37 (d, *J*_HH_ = 7.7 Hz, 1H; kynu), 8.62 (s, 1H; OH-kynu) ppm. ^31^P{^1^H} NMR (CDCl_3_ - 161.73 MHz): *δ* (ppm) 9.94 (t, 2P, *J*_PP_ = 39 Hz); –12.1 (t, 2P, *J*_PP_ = 39 Hz); –144.7 (sept, 1P, *J*_PF_ = 711 Hz). UV-vis (CH_2_Cl_2_, 5.0 × 10^–5^ M): *λ*/nm (*ε*/M^–1^ cm^–1^) 249sh (3.11 × 10^4^), 315sh (5.22 × 10^3^), 329 (7.10 × 10^3^), 344 (7.4 × 10^3^), 363 (4.01 × 10^3^). Molar conductivity [Λ_M_/(S·cm^2^·mol^–1^) in methanol: 102.0.

### 2.4. Interactions Studies

#### 2.4.1. CT-DNA

Measurements involving CT-DNA (calf thymus from Sigma-Aldrich) were carried out in a Trizma buffer (4.5 mM Trizma HCl, 0.5 mM Trizma base, and 50 mM NaCl, pH 7.4). The DNA concentration per nucleotide was determined by absorption spectrophotometric analysis using a molar absorption coefficient of 6600.0 mol^–1^·L·cm^–1^ at 260.0 nm [19].

#### 2.4.2. Circular Dichroism (CD) Experiments

CD spectra were recorded on a spectropolarimeter JASCO J-720 between 540 and 240 nm in a continuous scanning mode (200 nm·min^−1^). The final data are expressed in molar ellipticity (millidegrees). All of the CD spectra were generated and represented averages of three scans. Stock solutions of each complex were freshly prepared in DMSO prior to use. An appropriate volume of each solution was added to the samples of a freshly prepared solution of CT-DNA (50 *µ*M) in Trizma buffer to achieve molar ratios ranging from 0.05 to 0.5 DNA·drug^−1^. The samples were incubated at 37°C for 18 h.

#### 2.4.3. BSA-Binding Experiments

The protein interaction with complexes** 1** and** 2** was examined in 96-well plates used for fluorescence assays on a fluorimeter Synergy H1. BSA (2.0 *µ*mol·L^−1^) was prepared in Trizma buffer at pH = 7.4 (4.5 mM Trizma HCl, 0.5 mM Trizma base and 50 mM NaCl).

The inner-filter effect for ruthenium complexes was corrected by using

(1) $$\begin{array}{c} F_{corr}=F_{obs}e^{\left(A_{em}+A_{ex}\right)/2}, \end{array}$$

where *F*_corr_ and *F*_obs_ are the corrected and measured fluorescence intensity of protein, respectively. *A*_em_ and *A*_ex_ are the absorption values of the system at the excitation wavelength and emission wavelength of the complex, respectively.

Complexes were dissolved in sterile DMSO. For fluorescence measurements, the BSA concentration was kept constant in all samples, while the complex concentration was increased from 3.125 to 150 *μ*M, and quenching of the emission intensity of the tryptophan residues of BSA at 320 nm (excitation wavelength 280 nm) was monitored at different temperatures (300 and 310 K). The experiments were carried out in triplicate and analyzed using the classical Stern-Volmer equation

(2) $$\begin{array}{c} \frac{F_{0}}{F}=1+k_{q}\tau_{o}\left[\;Q\;\right]=1+K_{sv}\left[\;Q\;\right], \end{array}$$

where *F*_0_ and *F* are the fluorescence intensities in the absence and presence of quencher, respectively, [*Q*] is the quencher concentration, and *K*_sv_ is the Stern-Volmer quenching constant, which can be written as

(3) $$\begin{array}{c} K_{q}=\frac{K_{sv}}{\tau_{o}}, \end{array}$$

where *K*_*q*_ is the biomolecular quenching rate constant and *τ*_*o*_ is the average lifetime of the fluorophore in the absence of quencher (6.2 × 10^−9^ s) [20]. Therefore, (2) was applied to determine *K*_sv_ by linear regression of a plot of *F*_0_/*F* versus [*Q*].

The binding constant (*K*_*b*_) and number of complexes bound to BSA (*n*) were determined by plotting the double log graph of the fluorescence data using

(4) $$\begin{array}{c} log\frac{\left(F_{0}-F\right)}{F}=\log K_{b}+n\log\left[\;Q\;\right]. \end{array}$$

The thermodynamic parameters were calculated from the van't Hoff equation:

(5) $$\begin{array}{c} \ln K_{b}=\frac{\Delta H^{{}^{\circ}}}{RT}+\frac{\Delta S^{{}^{\circ}}}{R}, \end{array}$$

where *K*_*b*_ is analogous to the Stern-Volmer quenching constant, *K*_sv_ is at the corresponding temperature (the temperatures used were 300 and 310 K), and *R* is the gas constant, from which the Δ*H* and Δ*S* of the reaction can be determined from the linear relationship between ln⁡*K*_*b*_ and the reciprocal absolute temperature. Furthermore, the change in free energy (Δ*G*) was calculated from the following equation:

(6) $$\begin{array}{c} \Delta G^{{}^{\circ}}=-RT\ln K_{b}=\Delta H^{{}^{\circ}}-T\Delta S^{{}^{\circ}}. \end{array}$$

### 2.5. Human Cell Lines and Culture Conditions

Cells from the 4th through to the 12th passage were used. The different cell lines were maintained as monolayers in plastic culture flasks (25 cm^2^) containing HAM-F10 plus DMEM (1 : 1; Sigma-Aldrich) or only DMEM depending on the cell line, supplemented with 10% fetal bovine serum (Nutricell) and 2.38 mg·mL^–1^ Hepes (Sigma-Aldrich) at 37°C in a humidified 5% CO_2_ atmosphere. Antibiotics (0.01 mg·mL^–1^ streptomycin and 0.005 mg·mL^–1^ penicillin; Sigma-Aldrich) were added to the medium to prevent bacterial growth.

### 2.6. Cell Viability Assay

The screening for cytotoxic activity of cell lines was assessed using the Colorimetric Assay In Vitro Toxicology-XTT Kit (Roche Diagnostics). For the experiments, 1 × 10^4^ cells were seeded into microplates with 100 *µ*L of culture medium (1 : 1 HAM F10 + DMEM or alone DMEM) supplemented with 10% fetal bovine serum containing concentrations of complexes ranging from 12.5 to 1600 *µ*g·mL^−1^. Negative (no treatment), solvent (0.02% DMSO), and positive (25% DMSO) controls were included. Positive controls comprising cisplatin (Sigma-Aldrich, ≥98% purity) were included. After incubation to 36.5°C for 24 h, the culture medium was removed. Cells were washed with 100 *μ*L of PBS for removal of the treatments, after which they were exposed to 100 *μ*L of culture medium HAM-F10 without phenol red. Then, 25 *µ*L of XTT was added and incubated at 36.5°C for 17 h. The absorbance of the samples was determined using a multiplate reader (ELISA-Tecan-SW Magellan versus 5.03 STD 2P) at a wavelength of 450 nm and a reference length of 620 nm.

### 2.7. Statistical Analysis

Cytotoxicity was assessed using the IC_50_ response parameter (50% cell growth inhibition) calculated with the GraphPad Prism program, plotting cell survival against the respective concentrations of the treatments. One-way ANOVA was used for the comparison of means (*P* < 0.05). The selectivity index was calculated by dividing the IC_50_ value of the isolated compounds on GM07492-A cells by the IC_50_ value determined for human cancer cells.

## 3. Results and Discussion

### 3.1. Synthesis

**Figure 1:**
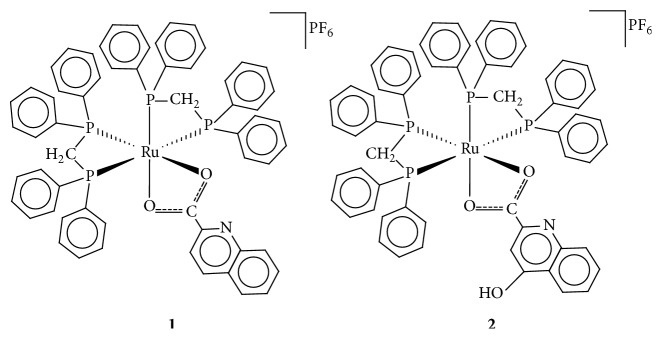
Structures of the ruthenium(II) compounds obtained in this work.

The simple reaction of the quinoline carboxylic acids with ruthenium(II) diphosphine precursor complex* cis*-[RuCl_2_(dppm)_2_] resulted in the products* cis*-[Ru(quin)(dppm)_2_]PF_6_** (1)** and* cis*-[Ru(kynu)(dppm)_2_]PF_6_** (2)**, Figure 1, by simple chlorido exchange under mild conditions.

### 3.2. Spectroscopical Characterization

The diamagnetic and monoelectrolytes compounds** 1**-**2** exhibited satisfactory microanalytical (C, H, and N) data. The ^1^H NMR spectra showed two broad signals due to the CH_2_ group of the dppm ligand close to 4.10 and 4.70 ppm [21]. The hydrogens of the phenyl groups (H_o_, H_m_ and H_p_) were observed as several multiplets between 6.26 and 7.86 ppm [21]. For complex** 1** three doublets of the quin ligand were observed at 7.91, 8.22, and 8.31 ppm while for complex** 2** one doublet at 8.37 ppm and two singlet signals at 6.80 and 8.62 ppm could be assigned to the kynu ligand [22]. Other hydrogens of the quin and kynu ligands were obscured by the signals corresponding to the hydrogens of the phenyl groups. The total number of hydrogens and proportion between the ligands dppm and quin or kynu was confirmed by the integral values for both complexes. In the ^31^P{^1^H} NMR spectra these complexes displayed a pair of triplets resonance signals, corresponding to two* trans*positioned phosphorus atoms and two phosphorus atoms* trans*positioned to oxygen atoms from carboxylate groups of the quinaldate and kynurenate anions, respectively. Triplets are observed at 9.70 and −12.2 ppm and at 9.94 and −12.1 ppm, respectively, for** 1** and** 2**, with the splitting pattern typical of an AX pattern [16]. These signals are downfield shifted when compared with the triplets signals for the* cis*-[RuCl_2_(dppm)_2_] that are observed at 0.64 and −25.3 ppm with *J*_PP_ = 36 Hz [17].

The IR spectrum displayed the typical asymmetric (*ν*_asym_) and symmetric (*ν*_sym_) carboxylate stretching frequencies at 1523 and 1455 cm^−1^ (Δ = 68 cm^–1^) and 1516 and 1454 (Δ = 62 cm^−1^), respectively, for** 1** and** 2,** confirming the presence of the quinoline carboxylate ligands coordinated in the chelating mode through the carboxylate to the metal center [23, 24]. The characteristic P-F stretch of the PF_6_^−^ counterion was seen at 840 and 557 cm^−1^ [23].

### 3.3. Circular Dichroism (CD) Experiments

**Figure 2:**
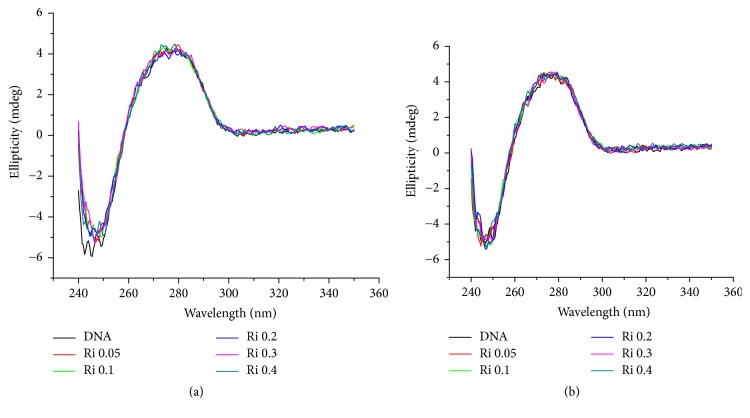
Circular dichroism (CD) spectra of CT-DNA incubated 18 h with complexes** 1** (a) and** 2** (b) at different [complex]/[DNA] ratios at 37°C.

The CD spectral technique is very sensitive for diagnosing changes in the secondary structure of DNA, resulting from drug-DNA interactions [25]. A typical CD spectrum of CT-DNA shows a maximum at 275 nm, due to the base-stacking and a minimum at 248 nm attributed to the right-handed helicity, characteristic of the B conformation [26]. Thus simple groove binding and electrostatic interaction of small molecules show less or no perturbation on the base-stacking and helicity bands, while intercalation enhances the intensities of both the bands stabilizing the right-handed B conformation of CT-DNA as observed for the classical intercalator methylene blue [27]. To determine if the ruthenium(II) complexes cause changes in DNA, CD spectra of CT-DNA with increasing concentrations of** 1** and** 2** were acquired, up to molar ratio drug·DNA^−1^ (Ri) = 0.4. As shown in Figure 2 significant changes were not observed indicating that these compounds were not able to intercalate DNA [28].

### 3.4. BSA-Binding Experiments

Serum albumin is the most abundant protein in plasma and is involved in the transport of metal ions and metal complexes with drugs through the blood stream. Binding to these proteins to complexes may lead to loss or conformational change in the protein subunit and provide paths for drug transportation. Bovine serum albumin, BSA (containing two tryptophans, Trp-134 and Trp-212) is the most extensively studied serum albumin, due to its structural homology with human serum albumin, HAS (one Trp-214). The BSA solution exhibits a strong fluorescence emission with a peak at 340 nm, due to the tryptophan residues, when excited at 280 nm [29]. Fluorescence quenching of BSA can occur by different mechanisms, usually classified as either dynamic or static quenching, which can be distinguished by their differing on temperature, viscosity, and lifetime measurements [30]. A dynamic quenching refers to the collisional process between the fluorophore and the quencher (in this case, ruthenium complexes) during the transient existence of the excited state. Dynamic quenching depends on diffusion, as higher temperatures result in high diffusion coefficient, and consequently, the constant quenching must also increase. In contrast, for the static quenching, an increase in temperature results in lower extinction values of the constants due to a fluorophore and quencher complex formation in the ground state [31].

**Figure 3:**
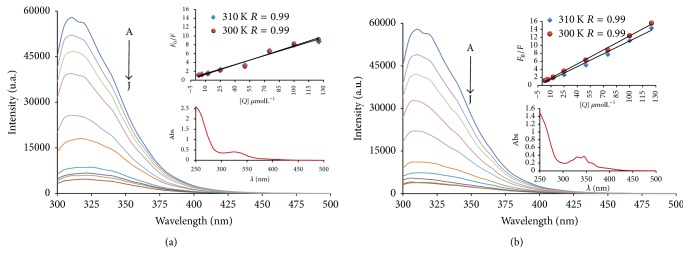
Fluorescence emission spectra of the BSA (2.5*µ*M *λ*_ex_ 280 nm) at different concentrations of complexes** 1** (a) and** 2** (b) at 300 K. Inset: Stern-Volmer plots showing tryptophan quenching in BSA at 300 and 310 K and UV-vis spectra of complexes** 1** and** 2**.

The Stern-Volmer equation (2) has been used to understand the nature of the quenching mechanism of BSA in the presence of complexes** 1** and** 2** at different temperatures [31].  Figure 3 shows the quenching of the BSA in the presence of the different concentrations of the complexes** 1** and** 2**.

In general, these complexes showed no significant variation *K*_sv_ with increasing temperature. However, the results of *K*_*q*_ have values greater than the maximum possible for a dynamic mechanism (2.0 × 10^10^ L·mol^−1^·s^−1^) [32] and in both cases were of the order 10^13^ M^−1^·s^−1^ (Table 1), which is 1000-fold higher than the maximum value possible for diffusion controlled quenching of various kinds of quencher to biopolymer. It is suggested that the suppression of BSA with these complexes is a static quenching mechanism. The decreasing of *K*_*q*_ with increasing temperature is in accordance with *K*_sv_ dependence on temperature.

In both cases, the obtained values of* n* indicate that the proportion between BSA-complex is equal 1 : 1, indicating that there is only one binding site in the BSA for each ruthenium complex, similar or equal to those reported before for other metal complexes [33–35]. Furthermore, values of *K*_*b*_ confirmed the moderated interaction force between complex-BSA [36–38] and the temperature is not significant. Thus, these complexes can be stored and carried by protein in the body. The interaction forces between drugs and biomolecules may include van der Waals interaction, hydrogen bonds, and electrostatic and hydrophobic interactions. The thermodynamic parameters Δ*G* (free energy change), Δ*H* (enthalpy change), and Δ*S* (entropy change) were calculated to evaluate the intermolecular forces involving the molecules of complex and BSA. The values for Δ*H* > 0 and Δ*S* > 0 imply the involvement of hydrophobic forces in protein binding, Δ*H* < 0 and Δ*S* < 0 correspond to van der Waals and hydrogen bonding interactions, and Δ*H* < 0 and Δ*S* > 0 suggest an electrostatic force [39]. Thermodynamic parameters (Δ*H* and Δ*S*) were calculated from the van't Hoff plots, (5); Δ*G* was estimated from (6). All the results are shown in Table 1.

The negative values of free energy, Δ*G*, suggested that the interaction process was spontaneous; the values entropy Δ*S* and enthalpy Δ*H* negative indicated that the hydrogen bonds and van der Waals forces are the more important interactions in the reaction [38].

### 3.5. Cytotoxicity Assays

**Figure 4:**
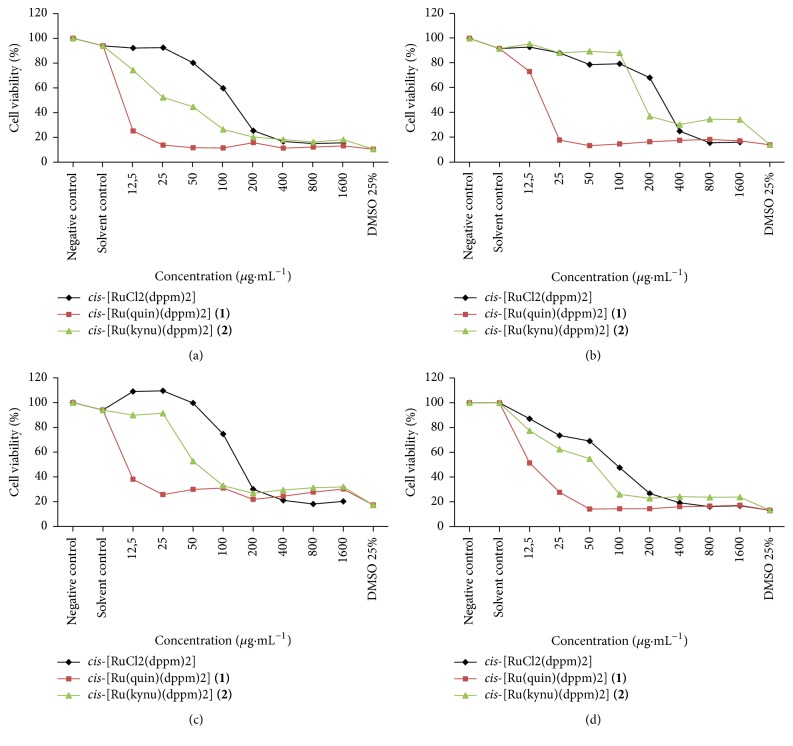
In vitro cytotoxicity activities of ruthenium complexes in HepG2 (a), MCF-7 (b), MO59J (c), and GM07492A (d) cells after exposure for 24 h. The concentration-effect curves were determined using XTT assay. Each data point is the mean ± standard error obtained from three independent experiments.

Ruthenium compounds** 1** and** 2**,* cis*-[RuCl_2_(dppm)_2_] and cisplatin were evaluated for their capability of inhibiting tumor cell growth in vitro using three human cell lines, HepG2, MCF-7, and MO59J. One nontumorigenic cell line (GM07492A) was also assayed in the same conditions in order to verify the selectivity of these compounds. The resulting concentration-effect curves obtained with continuous exposure for 24 h are depicted in Figure 4. A more convenient comparison of the cytotoxic potency (expressed as IC_50_ values) is listed in Table 2.

Overall sensitivities of the three tumor cell lines (Figures 4(a)–4(c)) and of the normal cell (Figure 4(d)) are more pronounced for complex** 1,** followed by complex** 2,** and the less active is the precursor* cis*-[RuCl_2_(dppm)_2_].

Complexes** 1** and** 2** have showed, in general, a moderate cytotoxicity against all the human tumor cell lines assayed with IC_50_ values ranging from 10.1 to 36 *µ*M, except for complex** 2** in MCF-7 cells that showed a very low cytotoxicity as shown in Table 2. Complex** 1** displayed higher activity than** 2** in all the cell lines assayed with IC_50_ close to 10 *µ*M. This nonselective activity of complex** 1** was not observed for complex** 2** that was almost inactive in MCF-7 cells. The selectivity index (SI) was very close to 1 (or smaller) for all the cell lines assayed indicating a lack of selectivity for both complexes. In the same experimental conditions the precursor complex* cis*-[RuCl_2_(dppm)_2_] was less active than the complexes** 1** and** 2** by factors ranging from 1.4 to 15.8. A similar increased in activity was also observed against the normal cell line GM07492A. This lack of selectivity was also observed for the* cis*-[RuCl_2_(dppm)_2_] for all the cell lines assayed and for cisplatin in the MCF-7 and MO59J cell lines. These data clearly indicate that exchanging two chlorido ligands by a bidentate quinoline carboxylate group turns the unity* cis*-[Ru(dppm)_2_] complex more cytotoxic, probably due to its higher solubility and disponibility in the culture medium. Interestingly, the presence of one OH-group in complex** 2** led to a significant decrease in cytotoxic activity, indicating that this may be a way to modulate the cytotoxic potency of this type of complex.

## 4. Conclusion

In this investigation two new ruthenium(II) complexes containing dppm and the quinaldate and kynurenate anions with formula* cis*-[Ru(quin)(dppm)_2_]PF_6_ and* cis*-[Ru(kynu)(dppm)_2_]PF_6_ were synthesized and characterized by elemental analysis and spectroscopic methods. Characterization data are in agreement with a* cis* geometry and a chelated coordination, through the carboxylate group, for the quinaldate and kynurenate ligands. Utilizing circular dichroism spectroscopy showed that these complexes lack the ability to intercalate DNA. On the other hand BSA-binding constants and thermodynamic parameters suggest spontaneous interactions with this protein by hydrogen bonds and van der Waals forces. The in vitro cytotoxicity activity assays indicate a moderate to high cytotoxicity against a panel of human tumor cell lines; however, these complexes lacks selectivity. Interestingly, modulation of cytotoxic potency can probably be done by exchanging the substituents of the quinolone carboxylate ligand and will be explored in future work.

## Figures and Tables

**Table 1:** Stern-Volmer quenching constant (*K*_sv_, L·mol^−1^), biomolecular quenching rate constant (*K*_*q*_, L·mol^−1^·s^−1^), binding constant (*K*_*b*_, L·mol^−1^), the number of binding sites (*n*), and Δ*G*^0^ (KJ·mol^−1^), Δ*H*^0^ (KJ·mol^−1^), and Δ*S*^0^ (J·mol^−1^·K) values for the complex-BSA system at different temperatures.

		*K*_sv_	*K*_*q*_	*K*_*b*_	*n*	Δ*G*^0^	Δ*H*^0^	Δ*S*^0^
		(×10^5^)	(×10^13^)	(×10^5^)				
**1**	300	0.77	1.10	4.77	1.2	−32.61	−87.78	−183.91
	310	0.75	1.10	1.53	1.0	−30.77		
**2**	300	1.24	1.79	3.25	1.1	−31.65	−50.16	−61.70
	310	1.00	1.50	1.70	1.0	−31.04		

**Table 2:** Inhibitory activity of ruthenium complexes and cisplatin against normal and tumor cell lines for 24 h incubation, expressed as IC_50_, *µ*g·mL^−1^ (*µ*M) and selectivity index (SI).

	Cell line						
	HepG2^a^	MCF-7^b^	MO59J^c^	GM07492A^d^	SI^1^	SI^2^	SI^3^
*cis*-[RuCl_2_(dppm)_2_]	102 ± 26 *(108 ± 26)*	180 ± 13 *(191 ± 13)*	126 ± 10 *(134 ± 10)*	62 ± 4 *(66 ± 4)*	0.61	0.34	0.49
**1**	11.8 ± 0.7 *(10.1 ± 0.6)*	14.2 ± 0.6 *(12.1 ± 0.5)*	13 ± 2 *(11 ± 2)*	11 ± 1 *(10 ± 1)*	0.93	0.77	0.85
**2**	31 ± 6 *(26 ± 5)*	162 ± 14 *(135 ± 11)*	44 ± 3 *(36 ± 2)*	45 ± 1 *(37 ± 1)*	1.45	0.28	1.02
cisplatin^e^	1.9 ± 0.2 *(6.3 ± 0.7)*	10 ± 1 *(34 ± 4)*	7 ± 1 *(22 ± 4)*	8 ± 1 *(26 ± 3)*	4.21	0.80	1.14

^a^Human hepatocellular carcinoma, ^b^human breast adenocarcinoma, ^c^human glioblastoma, ^d^normal human lung fibroblasts, and ^e^reference drug. SI^1^ = IC_50_ GM07492A/IC_50_ HepG2; SI^2^ = IC_50_ GM07492A/IC_50_ MCF-7; SI^3^ = IC_50_ GM07492A/IC_50_ MO59J.
